# Femtosecond Laser Fabrication of Engineered Functional Surfaces Based on Biodegradable Polymer and Biopolymer/Ceramic Composite Thin Films

**DOI:** 10.3390/polym11020378

**Published:** 2019-02-20

**Authors:** Albena Daskalova, Irina Bliznakova, Liliya Angelova, Anton Trifonov, Heidi Declercq, Ivan Buchvarov

**Affiliations:** 1 Institute of Electronics, Bulgarian Academy of Sciences, 72, Tsarigradsko Chaussee blvd., 1784 Sofia, Bulgaria; irbliznakova@abv.bg (I.B.); lily1986@abv.bg (L.A.); 2 Physics Department, Sofia University “St. Kliment Ohridski”, 5 J. Bourchier Blvd., BG-1164 Sofia, Bulgaria; a.trifonov@phys.uni-sofia.bg (A.T.); ivan.buchvarov@phys.uni-sofia.bg (I.B.); 3 Department of Basic Medical Sciences, Ghent University, De Pintelaan 185 6B3, 9000 Gent, Belgium; heidi.declercq@ugent.be

**Keywords:** femtosecond laser processing, functional surface, biopolymers, bioceramics, tissue engineering

## Abstract

Surface functionalization introduced by precisely-defined surface structures depended on the surface texture and quality. Laser treatment is an advanced, non-contact technique for improving the biomaterials surface characteristics. In this study, femtosecond laser modification was applied to fabricate diverse structures on biodegradable polymer thin films and their ceramic blends. The influences of key laser processing parameters like laser energy and a number of applied laser pulses (*N*) over laser-treated surfaces were investigated. The modification of surface roughness was determined by atomic force microscopy (AFM). The surface roughness (*R*_rms_) increased from approximately 0.5 to nearly 3 µm. The roughness changed with increasing laser energy and a number of applied laser pulses (*N*). The induced morphologies with different laser parameters were compared via Scanning electron microscopy (SEM) and confocal microscopy analysis. The chemical composition of exposed surfaces was examined by FTIR, X-ray photoelectron spectroscopy (XPS), and XRD analysis. This work illustrates the capacity of the laser microstructuring method for surface functionalization with possible applications in improvement of cellular attachment and orientation. Cells exhibited an extended shape along laser-modified surface zones compared to non-structured areas and demonstrated parallel alignment to the created structures. We examined laser-material interaction, microstructural outgrowth, and surface-treatment effect. By comparing the experimental results, it can be summarized that considerable processing quality can be obtained with femtosecond laser structuring.

## 1. Introduction

Regenerative medicine and tissue engineering now expands to practically all areas of healthcare. According to a new report by Grand View Research, Inc. (San Francisco, CA, USA) [1], the global tissue engineering market is expected to reach USD 11.53 billion by 2022. Reconstruction of bone tissue defects is a major challenge facing orthopedics, as treatment of skeletal defects has remained a demanding part of many reconstructive surgeries [2,3]. When the gold standard for bone grafting (autogenous bone) is inapplicable, bone tissue engineering is emerging as a possible solution. Recently, temporary “platforms” of various materials for seeding different types of cell cultures and improving cell adhesion, proliferation, and/or differentiation has been extensively researched. These scaffolds act as a support structure for the cell attachment and growth into bone tissues and must have adequate mechanical properties [4].

The main representatives of biomaterials used for design of tissue scaffolds models are made from three primary types of materials—polymers, metals, and ceramics. The requirements for a successful implant designed from biomimetic material in the human body are to possess specific properties that will prevent the rise of inflammation process triggered in living tissue. Furthermore, the demand of scaffolds design is to retain porosity with sizes > 100 µm [5], which is necessary for successful cell adhesion and expansion. The origins of human tissues content are complex materials that possess anisotropic characteristics. They differ in the distribution of various components, such as collagen, elastin, and hydroxyapatite present in the human body. Biomaterial scaffolds, which possess specific characteristics such as biocompatibility, porosity, and biodegradability, could be used as a template for tissue engineering [6,7]. Natural polymer materials are widely employed in tissue regeneration due to their unique biomimetic, biodegradable, and tribological characteristics. Composites comprised of calcium phosphates and natural biopolymers are widely used as biomaterials, as they meet the above-mentioned requirements for successful platforms for bone tissue repair and engineering [8,9,10,11,12]. Chitosan (Ch) is a natural polysaccharide derived from the *N*-deacetylation of chitin—the main component of crustacean exoskeletons. Because of its low toxicity, tunable biodegradability rate, bioresorbability, and high mechanical strength, it is being employed in wound healing of diverse injured tissues [13,14]. Therefore, the blending of chitosan with diverse polymers is a relevant approach to achieving scaffolds with good physical properties. Chitosan is considered to be a promising material exploited in biomedical applications such as nerve regeneration, bone disease treatment, ophthalmic biomaterial (contact lenses), and drug delivery [14,15,16]. It is used in composition with various biomaterials such as poly-ε-caprolactone (PCL), polylactic acid (PLA), and poly(lactic-*co*-glycolic acid) (PLGA) to produce scaffolds for diverse applications in tissue engineering. However, its application lacks effective cells binding (cell adhesion that influences cellular activity) [14]. To overcome this limitation, diverse methods for altering its porosity are applied, which include conventional chemicals or physical techniques such as solvent casting, particulate leaching, gas foaming, phase separation, and freeze drying. However, these methods do not provide precise pore size, geometry, interconnectivity, and spatial distribution of the pores [6], which are all crucial factors for the scaffold creation and cells seeding. In addition, the presence of residual traces of solvent may lead to risks of cells toxicity and carcinogenicity [17].

The most common bioactive materials that are used in bone and dental tissue engineering are bio ceramics such as zirconia (ZrO_2_), hydroxyapatite (HAp) and calcium phosphates ceramics (CaP). The major drawback of these materials is related to their brittle character and reduced bioactivity. On the other hand, polymers (such as chitosan) can be easily fabricated into complex shapes and porous structures. Biopolymers possess low toxicity and tunable biodegradability and bioresorbability rates. Thus, a combination of the best properties of bioceramics with biodegradable polymers is employed to achieve improved mechanical and biological features of engineered scaffolds.

Ceramic materials have been applied in various combinations with biopolymers such as collagen, chitosan, and fibrinogen for development of composite materials employed, especially in bone tissue applications [7,10,11,12,18,19,20,21,22]. Improving the longetivity of bone implants by incorporating the ceramic component enhances their physiological properties. For example, coating the cementless endoprostheses with a layer of plasma-sprayed hydroxyapatite promotes bone cells in growth and fosters the implant connection to the cortical bone matter [9].

Hydroxyapatite (HAp) is the main content of bone tissue. The incorporation of HAp as a source material for production of bone scaffolds is prerequisite since it is known for its excellent cellular affinity [8,9,10,11,12]. However, it has drawbacks expressed in insufficient mechanical stability. 

Because of its excellent mechanical properties, biocompatibility, and chemical stability, Zirconia (ZrO_2_) based ceramics attracted significant interest for the fabrication of bone tissue scaffolds [18,19]. The incorporation of ZrO_2_ into chitosan scaffold is believed to enhance the osteogenesis process. ZrO_2_ is bio-inert ceramic, and therefore it suffers from reduced cellular attachment [23].

The combination of polymers with bioceramics results in fabrication of bioactive matrices that stimulates tissue development with enhanced strength [8,9,10,11,12,20,21,22,23,24,25].

A few studies have reported functionalization of chitosan by mixing it with ceramic materials. For example, the groups of Wang, Muzzarelli, and Scheinpflug [26,27,28] reviewed, in detail, diverse approaches for applying polymer coatings or creating polymer-bioceramic microstructures that imitate the composite arrangement of bone and could be applied for bone regeneration.

The inclusion of ZrO_2_ and HAp to chitosan thin films helps to prepare the substrate with improved cellular activity properties, which will enhance the bone regeneration [29].

Nanotechnology has been increasingly utilized to enhance bone tissue engineering strategies [30,31]. The addition of silver nanoparticles to the thin films improves their qualities even more, as silver nanoparticles are well known for their antibacterial properties. [32]. Nanosilver has gained considerable application in recent years due to its sustained silver ions release. Thus, the antibacterial effect, by reducing cell toxicity, could be significantly improved by the incorporation of Ag nanoparticles into the composite films. As a continuation of our recent examinations on the processing of biopolymer thin chitosan films [33,34,35,36,37], we focused towards the creation of patterning designs and characterization of thin films of pure chitosan and its composite scaffolds from HAp/ZrO_2_ for achievement of successful platforms for bone tissue repair and engineering characterized not only by high biocompatibility, biodegradability, and affinity to proteins, but also by good chemical and dimensional stability, mechanical strength, and toughness, which will improve the tissue engineering applications of the scaffolds.

For creation of tissue engineered scaffolds, a set of chemical and physical methods are used for the production of a biomaterial matrix. In most cases, they are designed from polymers or their blends onto which the cells are seeded. Surface topography of the biomaterial has been shown to affect cellular mobility, which is crucial to achieving optimal functionalization of the biomaterial. Surface nanostructuring via the creation of homogeneous nanopatterns on the surface of polymers provides possibilities for nanofabrication of functional polymer materials. Rebollar et al. demonstrated the application of laser-based methods (laser induced periodic surface structures (LIPSS)) for fabrication of high spatial resolution patterning of soft polymeric material ((PET), poly (trimethylene terephthalate) (PTT) and polycarbonate bisphenol). They found that by tuning the period and shape of the LIPSS, control over characteristics of the superficial structures could be obtained. More specifically, the rippled polymer films could be employed as substrates for cell culture/alignment [38]. Another application of surface structuring was demonstrated by Wang et al. for fabrication of in-plane and multi-layer 3D micro-supercapacitors (MSCs) based on laser carbonization of polyimide (PI) sheets. They demonstrated that via the creation of hierarchical porous structures and the appropriate heteroatom nitrogen/oxygen doping, superior performance of carbon-based MSCs fabricated by laser direct writing were obtained. The fabricated 2-layer and 3-layer stacked MSCs demonstrated enhanced specific capacitance with an increase from 37.2 mF/cm^2^ and 42.6 mF/cm^2^ compared to other carbon material-based MSCs. [39].

Via selection of suitable biomaterials and application of additional surface treatment and coatings to the designed scaffolds, improvement of biocompatibility properties could be achieved.

The influence of laser treatment on self-standing chitosan films and the composites with HAp/ZrO_2_ over porosity, microstructure, and chemical composition of the biopolymer and biopolymer/ceramic samples were investigated. In this study, we present the processing of chitosan and chitosan/HAp/ZrO_2_ composite thin films and the development of surface structures based on femtosecond laser irradiation. We experimentally demonstrated that under exposure to a selected set of parameters of femtosecond laser radiation, we obtained a patterned surface with tunable surface roughness. The results from the processing of the chitosan/ZrO_2_ blends showed the formation of LIPSS at the bottom of ablation craters under specific laser irradiation parameters (*N* = 1 and 2). In this study, we focused our attention towards topography of the materials in order to mimic the surface properties similar to natural body tissues.

The microstructured thin films were investigated by scanning electron microscopy (SEM), atomic force microscopy (AFM), fourier-transform infrared spectroscopy (FTIR), X-ray photoelectron spectroscopy (XPS), and X-Ray Diffraction (XRD) analyses.

## 2. Materials and Methods

### 2.1. Sample Preparation

Chitosan (medium molecular weight), hydroxyapatite powder (with particle size 10 µm, ≥ 100 m_2_/g), and nanoZrO_2_ (particle size < 100 nm) were purchased from Sigma-Aldrich^®^ (Munich, Germany). The chitosan powder was dissolved in a 0.5 mol/L CH_3_COOH solution and dH_2_O and heated to 50 °С under continuous stirring for 2.5 h. HAp was added to deionized water and stirred for approximately 2 h without heating. The two solutions were mixed together in diverse ratios of Ch/HAp. To the prepared ratios between Ch and HAp, 1% ZrO_2_ (*w*/*v*) was added and stirred without heating for 12 h before dripping. A solution of silver nanoparticles AgNps–10% Np (*v*/*v*) was added to the prepared ratios between Ch and HAp and stirred without heating for 1 h before dripping.

The biopolymer/ceramic composites were spread over 20 mm × 20 mm glass slides and allowed to dry at room temperature before irradiation by femtosecond laser pulses.

The samples were divided into two groups and treated with femtosecond laser radiation:

(1) Pure chitosan thin films.

(2) Different percentage of composite blends of chitosan (Ch)/HAp/ZrO_2_ thin films.

### 2.2. Experimental Setup

A femtosecond Ti:sapphire laser (Integra-C, Quantronix, Hamden, CT, USA) emitting a central wavelength of 800 nm was used. The pulse duration was 130 fs at 1 kHz repetition rate. The laser system possessed a possibility for variation of the repetition rate ν (from 25 Hz to 1 KHz) via delay generator. The number of laser shots (*N*) was controlled by computer-driven fast mechanical shutter synchronized from controlling software. The laser beam was focused using a lens with a focal length of 20 cm at normal incidence on the sample surface. It was estimated that the laser spot diameter on the surface of the sample was 50 µm.

**Figure 1:**
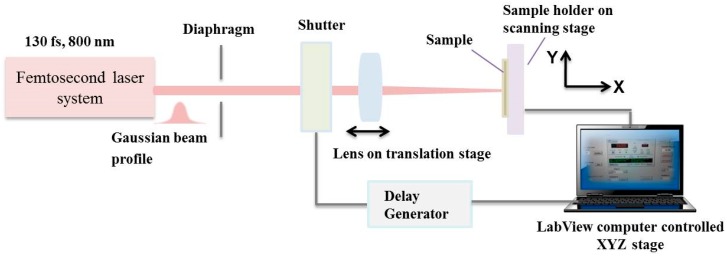
Optical setup for femtosecond laser modification of composite biomaterial structures.

A complete set of parameters was selected to deduce the key variables (laser energy, pulse number, overlapping between separate pulses), which effected the morphology, average roughness, and chemistry of the treated areas. Translation was achieved between separate sets of fired laser pulses so that the laser irradiated zone received a defined number of shots. The number of laser pulses (*N*) was varied from 1 to 100 to examine their influence on morphological change. A schematic of the experimental setup is shown in Figure 1.

The sample was positioned on a motorized xyz translation stage where the sample surface was placed perpendicular to the propagation direction of the incident laser radiation.

### 2.3. Morphological and Topography Examination of Textured Biofilms via SEM, Energy-Dispersive X-ray Spectroscopy (EDX), and Atomic Force Microscopy (AFM)

The morphology of the pure chitosan biofilms and their ceramic composites was analyzed using SEM-TESCAN/LYRA/XMU (TESCAN ORSAY HOLDING, a.s., Brno, Czech Republic). The samples were graphene sputtered. Several images were taken at laser treated and non-treated zones for each sample. Energy dispersive X-ray spectroscopy (EDX) was used to analyze the elemental composition of composite thin film surfaces before and after laser irradiation with an operational voltage of 20 kV. Each sample was measured at two positions inside the modified zone.

Roughness was measured by means of an AFM-Bruker Dimension Icon with Scan-Asystin several areas of the treated zones; average values of surface roughness were obtained to characterize the surface after the laser irradiation. In the case of surfaces treated by laser, the AFM tip ran the samples in the direction of the textures.

### 2.4. FTIR Analysis

In order to gain detailed information about the phase transformations and chemical bonds alterations in the samples, IR spectra of the prepared biofilms before and after laser treatment were obtained using FTIR spectrophotometer (IR Affinity-1, Shimadzu, Kyoto, Japan). The measurements were taken in transmittance mode, and the range recorded was from 4500 to 500 cm^−1^.

### 2.5. XPS Analysis

X-ray photoelectron spectroscopy (XPS) was performed by means of an AXIS Supra electron spectrometer (Kratos Analytical Ltd., Manchester, UK). XPS method enables high-resolution chemical analysis of polymer films. An evaluation of the surface chemistry of chitosan films after laser processing with a diverse number of applied laser pulses was obtained.

### 2.6. XRD Analysis

The surfaces of the samples after laser treatment were examined by XRD using PANalytical-Empyrean system (Malvern Panalytical, Malvern, UK) equipped with a Cu tube and a PIXcel3D detector. The experimental condition was: 40 kV, 40 mA, exposition 100 s, step 0.03 degree. Software High Score Plus (Malvern Panalytical, Malvern, UK) was used to analyze the obtained X-ray diffraction data. 

### 2.7. Cell Seeding, Culture, and Staining on Laser-Textured Samples

The arrangement of living cells on the patterned substrates was achieved via femtosecond laser texturing. The main groups of material used for cellular studies are classified in Table 1.

To sterilize the samples, they were placed under a UV lamp of 15 W (Sylvania, London, UK; 254 nm wavelength) at a distance of 45 cm and irradiated for 30 min. Mouse calvaria osteoblasts (MC3T3) and human adipose derived mesenchymal stem cells (ADSCs) (CryoSave, The Cell Factory, Niel, Belgium) were cultured in DMEM Glutamax (Gibco™; Invitrogen, Thermo Fisher Scientific, Waltham, MA, USA) supplemented with 10% fetal calf serum and 1% penicillin/streptomycin in a humidified atmosphere at 37 °C and 5% CO_2_. MC3T3 and ADSCs were seeded on laser-textured samples or on controls (non-textured samples or tissue culture plastic) in a 24-well plate. Densities of 100,000 cells and 40,000 cells per sample were used for MC3T3 and ADSCs, respectively. Cell adhesion and proliferation were evaluated after 1 and 6 days.

Live/dead staining with calcein AM/propidium iodide was performed to evaluate cell viability, cell attachment, and cell morphology. After rinsing the samples, the supernatant was replaced with 1 mL phosphate buffered saline (PBS) supplemented with 2 µL (1 mg/mL) calcein AM (Anaspec, Fremont, CA, USA; 89201) and 2 µL (1 mg/mL) propidium iodide (Sigma-Aldrich; P4170). After 10 min incubation in the dark at room temperature, the samples were washed with PBS and evaluated with a fluorescence microscope (Olympus IX 81, Olympus, Bridgeport, CT, USA).

## 3. Results

### 3.1. Femtosecond Laser Fabrication of Chitosan Biofilm Microporous Structures

**Figure 2:**
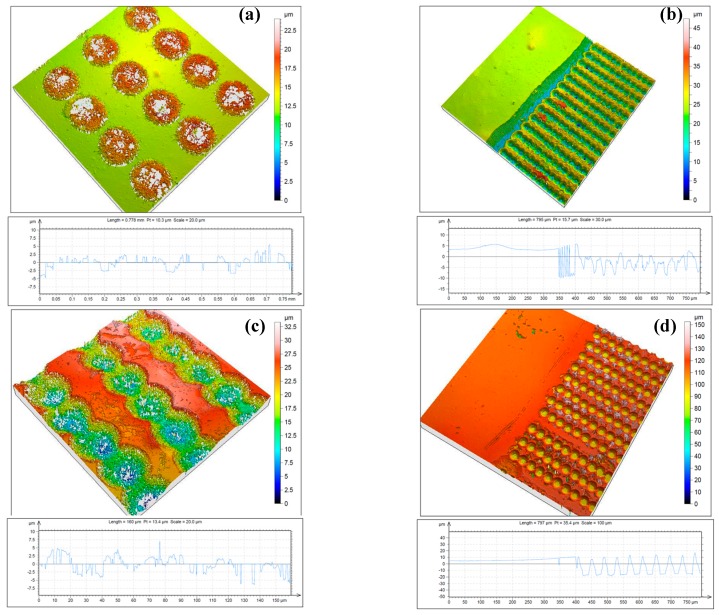
Three-dimensional topography and cross section of thin chitosan films irradiated by femtosecond (fs) laser, λ = 800 nm, τ = 130 fs, obtained by confocal microscopy: (**a**) *N* = 2, *I* = 7.9 × 10^14^ W/cm^2^; (**b**) *N* = 5, *I* = 7.9 × 10^14^ W/cm^2^; (**c**) *N* = 2, *I* = 1.5 × 10^15^ W/cm^2^; (**d**) *N* = 5, *I* = 1.5 × 10^15^ W/cm^2^.

Figure 2 shows a confocal microscopy image of structures fabricated by femtosecond laser irradiation for different surface treatments of chitosan thin films deposited on a cover glass.

The spots presented in Figure 2 were created in a series of irradiations with an increasing number of laser pulses, *N* = 2 and 5. For *N* = 2, there was a clearly detectable formation of sponge-like creation (Figure 2a) over the surface baseline. As *N* increased further to *N* = 5, the material from the surface began to ablate (Figure 2d). The optimal conditions to obtain the best quality of the fabricated “microfoam-like” structures were determined for a low number of applied laser pulses and low and intermediate laser intensities. At an increasing number of applied laser pulses (*N*), the structures porosity started to deviate, and removal of material was initiated, leading to crater formation.

These results demonstrated that microporous structures were fabricated by femtosecond laser irradiation for specifically selected exposition parameters (*N* = 2, *I* = 7.9 × 10^14^ W/cm^2^, Figure 2a). A clear difference in the morphological patterns of the chitosan surfaces was observed. Figure 2b,c) shows confocal microscopy images of chitosan films irradiated with increased laser intensity at two selected numbers of applied laser pulses (*N* = 2 and *N* = 5), which corresponded to the surface treatment that had the most suitable application in clinical use of the implant’s surface. In the case of treatment with *N* > 2, a formation of cavities through the removal of material by laser was observed, and destruction of microfoam started to take place. It was observed that a surface with uniform roughness and a creation of self-induced porosity due to the nature of non-contact interaction with fs laser radiation and the thin biopolymer films could be obtained under irradiation with a careful selection of a number of applied laser pulses (*N*~1 or 2) and a laser intensity (*I*) not exceeding *I* = 3.1 × 10^15^ W/cm^2^ [35].

### 3.2. AFM Topography Chitosan Biofilms

**Figure 3:**
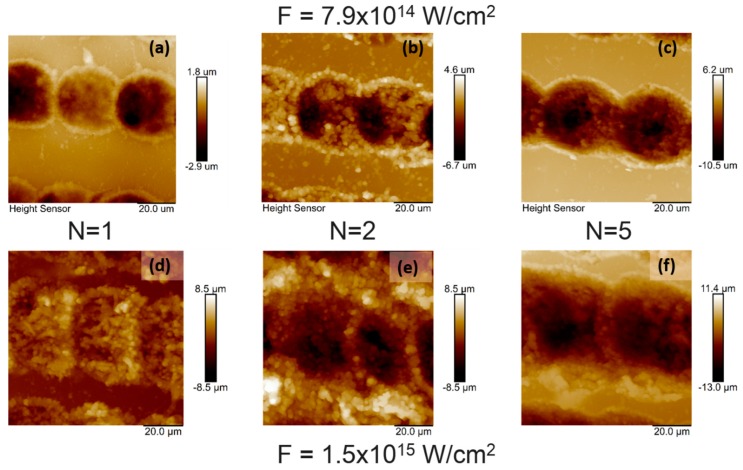
Atomic force microscopy (AFM) topography images of chitosan thin film over an area of 20 × 20 µm^2^ irradiated with fs laser at λ = 800 nm, τ = 130 fs at different *I* and *N*: (**a**) *I* = 7.9 × 10^14^ W/cm^2^, *N* = 1, *R*_rms_ = 0.780 µm; (**b**) *I*= 7.9 × 10^14^ W/cm^2^, *N* = 2, *R*_rms_ = 2. 266 µm; (**c**) *I* = 7.9 × 10^14^ W/cm^2^, *N* = 5, *R*_rms_ = 2. 430 µm; (**d**) *I* = 1.5 × 10^15^ W/cm^2^, *N* = 1, *R*_rms_ = 1.617 µm; (**e**) *I* = 1.5 × 10^15^ W/cm^2^, *N* = 2, *R*_rms_ = 2.272 µm; (**f**) *I* = 1.5 × 10^15^ W/cm^2^, *N* = 5, *R*_rms_ = 2.480 µm.

Figure 3 shows the topography image of irradiated chitosan thin film at a diverse number of applied laser pulses and at two energy settings obtained over an area of 20 × 20 μm^2^. The root mean-square roughness (*R*_rms_) at the irradiated zones deviated in relation to an increasing number of applied laser pulses and applied energy, with root mean-square roughness (*R*_rms_) increasing from 0.780 to 2.480 µm.

The images in Figure 3 show that pure chitosan thin films had distinct surface topography structures produced by different laser expositions. AFM images of chitosan biofilms revealed porous structures with a formation of craters on chitosan layers at higher intensities, which influenced the morphology via producing a rougher surface. The surface roughness deviation was estimated and related to changes due to the laser energy increase and increment of applied *N*. 

### 3.3. XPS Chracterization of Pure Chitosan Thin Films

**Figure 4:**
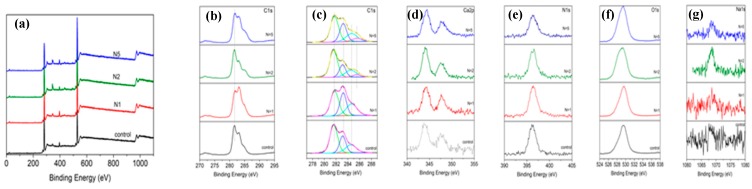
X-ray photoelectron spectroscopy (XPS) spectra of pure chitosan thin films (**a**) non-treated and laser irradiated at *I* = 7.9 × 10^14^ W/cm^2^ for *N* = 1, 2, 5, 10; (**b**)–(**g**) resolved peaks of C_1s_, C_1s-_peak fitting, Ca_2p_, N_1s_, O_1s_, Na_1s_.

The results of the XPS spectra analysis for pure chitosan films are shown in Figure 4. To compare the effect of laser treatment on the bonding strength, the examined material was separated in relation to applied laser conditions. All spectra were compared to non-irradiated chitosan surfaces. 

As seen from the XPS analysis in Figure 4, all six spectra exhibited peaks, showing binding energies at C_1s_, Ca_2p_, N_1s_, O_1s_, and Na_1s_.

The C_1s_ peak was divided into four peaks 281.7, 283.3, 284.7, and 285.45 eV corresponding to C–C/C–H bond and C–NH/C–NH_2_ (Figure 4b,c). After femtosecond laser treatment with an increasing number of pulses (*N* = 1–5), the C–C bond remained almost unchanged, and C–NH/C–NH_2_ appeared more pronounced when compared to the chitosan control. At 347.4 eV, a Ca_3_(PO_4_)_2_ was present. The N_1s_ spectrum exhibited a peak at 396.2 eV (Figure 4d), and the nitrogen signal arose from the chitosan amino groups and indicated a presence of chitosan in the surface layer. Another pronounced peak appeared at 529.4 eV, which could be assigned to hydroxyl groups (–ОН^−^) of O_1s_ line (Figure 4e). The intensity of the XPS peaks for chitosan did not change when increasing the number of applied laser pulses. These results indicated that the amount of characteristic components of chitosan did not decrease upon increasing *N*.

### 3.4. Surface Analysis via SEM of Chitosan/Ceramic Composite Films

**Figure 5:**
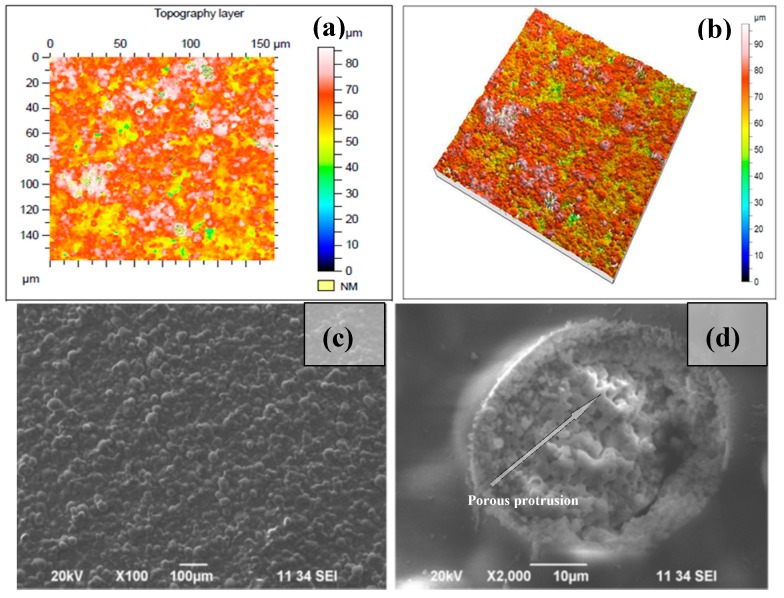
Thin 30/70% Ch/HAp biocomposite layers: (**a**) 150 µm × 150 µm area topography image, (**b**) 3-D topography confocal image of non-treated surface; (**c**) SEM image of non-treated surface; (**d**) laser irradiated surface with *N* = 1, *I* = 7.9 × 10^15^ W/cm^2^.

**Figure 6:**
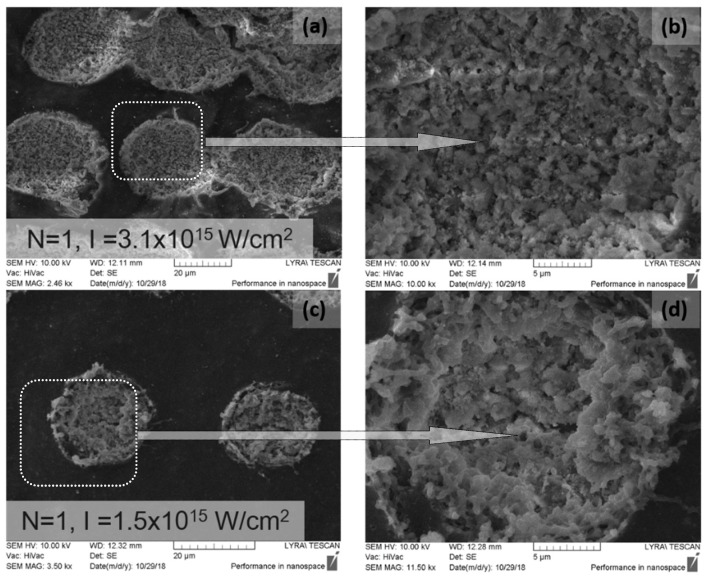
SEM images of 70/30% Ch/Hap + 1% ZrO_2_ thin films treated with fs laser radiation at (**a**) *N* = 1, *I* = 3.1 × 10^15^ W/cm^2^, (**b**) and (**d**) close-up of laser treated circular area, (**c**) *N* = 1, *I* = 1.5 × 10^15^ W/cm^2^.

**Figure 7:**
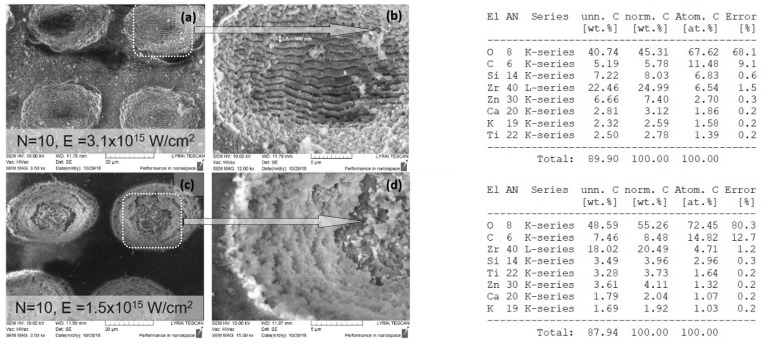
SEM images showing structures formed in 70/30% Ch/Hap + 1% ZrO_2_ due to irradiation with *N* = 10 at two laser intensities (**a**) *I* = 3.1 × 10^15^ W/cm^2^ (**c**) *I* = 1.5 × 10^15^ W /cm^2^; (**b**) and (**d**)—magnified view of treated zones; table (upper right): Energy dispersive X-ray (EDX) spectra of elements obtained after laser treatment at *I* = 3.1 × 10^15^ W/cm^2^; table (bottom right): Energy dispersive X-ray (EDX) spectra of elements obtained after laser treatment at *I* = 1.5 × 10^15^ W/cm^2^.

SEM images of the surface morphology for diverse surface treatments are presented in Figure 5, Figure 6, and Figure 7. Pure chitosan thin films possess a smooth surface. When blended with Hydroxyapatite, the surface morphology changed and showed a topography composed of granules (Figure 5a). There was a clear difference in the micropatterns of the Ch/HAp biocomposite layers after laser treatment with the formation of a porous area in the interaction zone with grain structures. In Figure 5b,c are SEM micrographs of the samples before and after laser treatment.

SEM micrographs of composite Ch/HAp layers reinforced with 1% ZrO_2_ revealed the presence of porous granular with nanoscale protrusion architecture Figure 6d. SEM analysis showed the development of granular morphology with porous formation on the sample surface at both laser conditions.

Comparing the different modifications and morphology obtained via laser treatment allowed a higher degree of roughness on the surface. Closer analysis of laser treated surfaces on the middle part of the crater (Figure 6b,d) showed granular structures, which are crucial for cellular interconnectivity, expansion, and proliferation.

Increasing the laser intensity triggers the formation of a deep crater, and at *N* = 10, *I* = 3.1 × 10^15^ W/cm^2^, glass substrate was reached, creating laser induced periodic surface structures LIPSS on the glass slide, as seen in Figure 7a,b. The period of LIPSS on glass substrate (Figure 7b) is Λ = 900 nm.

When the number of laser pulses (*N*) increased to 100, removal of the whole layer was observed. 

**Figure 8:**
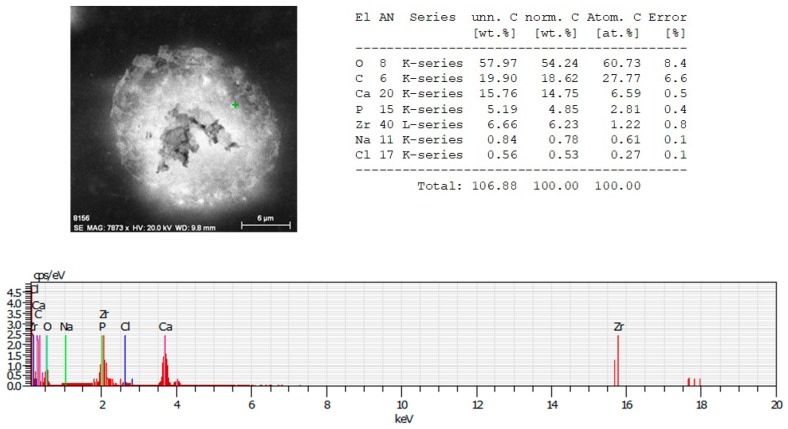
EDX spectrum of microstructure fabricated by femtosecond laser irradiation with *N* = 1, *I* = 1.5 × 10^15^ W/cm^2^ of thepolymer/ceramic composite—70% Ch/30% HAp/ZrO_2_. Table (upper right): EDX spectra showing the atomic percentages of O, C, Ca, P, Zr, Na, Cl.

EDX analysis of irradiated with *N* = 1, *I* = 1.5 × 10^15^ W/cm^2^, 70/30% Ch/Hap + 1% ZrO_2_ matrix, revealed the presence of Zirconium (Zr), Calcium (Ca), and phosphorus (P), which are common chemical elements present in the composition. The data showed a reduced amount of Zr element (Figure 8) when the surface was treated with a single pulse (*N* = 1) in relation to the treatment with *N* = 10, Figure 7. 

**Figure 9:**
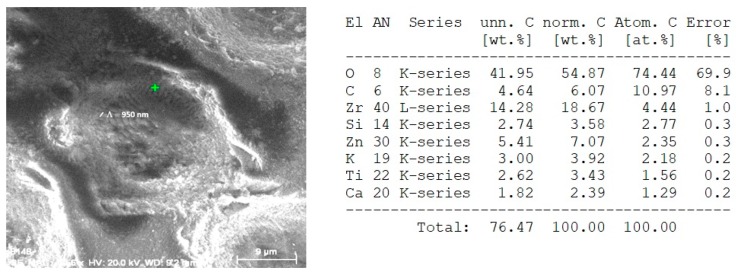
EDX analysis of 70% Ch/30% HAp/ZrO_2_ substrate treated with *N* = 10, *I* = 7.9 × 10^15^ W/cm^2^; table (right): EDX analysis showing the atomic percentages of O, C, Zr, Si, Zn, K, Ti, Ca.

In contrast to the observation of the morphological changes discussed above, we observed a limited interval where the creation of LIPSS in the thin composite layer was observed before the onset of full material ablation (Figure 9) when the number of laser pulses increased up to 10 and the laser intensity was 7.9 × 10^15^ W/cm^2^.

The LIPSS on Figure 9 were formed on the composite thin films, which was evidenced also by the acquired EDX analysis where a clear presence of Zr—14.28 wt.%, C—4.64 wt.%, and O—41.95 wt.% components from 70% Ch/30% HAp/ZrO_2_ thin films were detected. However, the formation of LIPSS was seen only at the edges of the spot formation close to the glass substrate with a periodicity close to the laser irradiation wavelength (Λ = 950 nm), as seen in Figure 9. Evidently, the glass substrate was contributing to the efficient feedback mechanism for LIPSS formation.

It was found that the periodic surface structures described as “ripples” formed preferentially for *N* = 5 to 10. They were parallel to the laser polarization direction. The formation mechanism of LIPSS was described with the optical interference between the incident wave and a surface scattered electromagnetic wave. LIPSS were fabricated for materials with low linear absorption coefficients. LIPSS were formed at a certain number of laser pulses, inducing nano-roughness. This affected the surface morphology by creating a path for the optimization of scaffold surface properties.

### 3.5. Fourier Transform Infrared Spectra for Laser Modified Chitosan Composite Films

The spectra of Ch/HAp/ZrO_2_ composites showed a mixture of characteristic transmittance due to the groups listed in Table 2.

**Figure 10:**
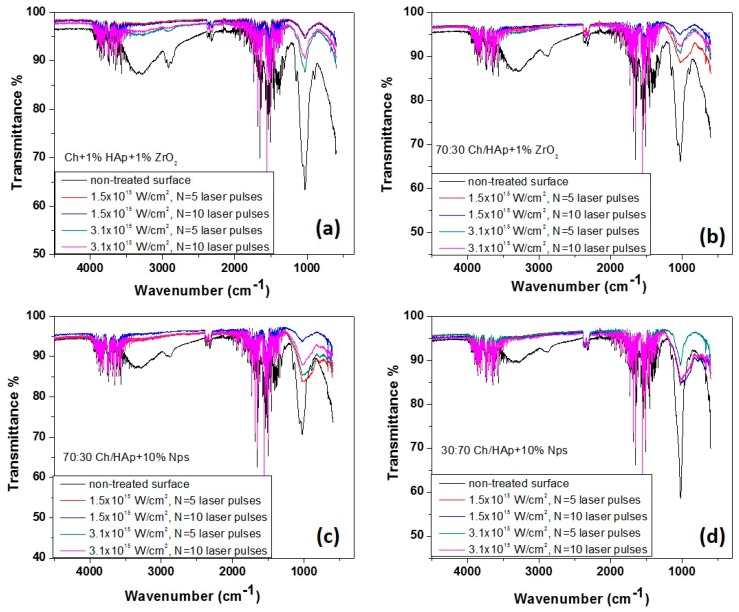
FTIR spectroscopy of chitosan/ceramic blends after laser irradiation at *N* = 5 and 10, and *I* = 1.5 × 10^15^ W/cm^2^, *I* = 3.1 × 10^15^ W/cm^2^. (**a**) Ch + 1% HAp + 1% ZrO_2_; (**b**) 70:30 Ch/HAp + 1% ZrO_2_; (**c**) 70:30 Ch/HAp + 10% Nps; (**d**) 30:70 Ch/HAp + 10% Nps.

The results illustrated significant differences in the spectra measured from the non-irradiated and laser-processed surfaces of the composite substrates. First, the FTIR spectra exhibited equal numbers and positions of the various transmittance peaks but with a major difference in the intensity of the peaks from the laser treated zones. The shapes of the spectra from both processed areas (*N* = 5 and *N* = 10 pulses) were similar (Figure 10). In the four cases, spectrum from the non-treated surface of chitosan blends exhibited one characteristic group between 3400 and 3000 cm^−1^. It corresponded to hydroxyl groups stretching in chitosan. The band between 2990–2880 cm^−1^ was attributed to –CH “backbone” vibrations, while the peaks near 1650 cm^−1^ corresponded to C=O stretching and =N–H vibrations of amide structures I and II, which were observed in all samples (laser treated and untreated). Another peak noticed at about 1267 cm^−1^ from the non-irradiated material surface was characteristic of the =N–H amide III structure. A maximum at about 1400 cm^−1^ was assigned to –CH_3_ and –CH_2_ groups in plane deformation vibrations [40,41]. The groups identified at about 1030 to 1020 cm^−1^ are commonly associated with the stretching of glucosamine in the chitosan structure. The transmittance maxima observed in the range of about 1000 cm^−1^ and 600–500 cm^−1^ were attributed to the stretching and flexing vibrations of the PO_4_^3−^ group. These peaks were much more pronounced in the non-irradiated samples in all of the chitosan blends examined, and from the irradiated ones, it was with higher intensity in the case of *N* = 5 and *I* = 1.5 × 10^15^ W/cm^2^. The area between 1130–1150 cm^−1^ was identified by P=O stretching vibrations [38].

The presence of nanoparticles was not detected. The observed FTIR peak in the region of 498–502 cm^−1^ was attributed to the vibration modes of ZrO_3_^2−^ groups, which confirmed the formation of the ZrO_2_ structure. One band at 600 cm^−1^ was assigned to the Zr–O–Zr bendings [42,43]. In general, the profile of all peaks for the case of femtosecond irradiated surfaces for all presented chitosan/hydroxyapatite concentrations coincided, as the only difference was in the intensity of the lines with a tendency to decrease with the increment of laser intensity and a number of the applied pulses. The main effect of the interaction of laser radiation was characterized by a significant reduction in the maxima associated with the saccharide ring of chitosan, which almost disappeared due to the increase in the laser intensity and the number of laser pulses. This was explained by the greater accumulation of energy in the interaction zone, which was related to the change of the polymer structure in composite biopolymer/ceramics, which in turn led to a change in the structure of the biopolymer. In a similar type of experiment from our earlier studies, we found that the threshold modification for a chitosan specimen was much lower compared to chitosan/hydroxyapatite composites [33], as was seen here for the higher concentration of HAp Figure 10d.

### 3.6. XRD Analysis of Composite Chitosan/Ceramic Films

The XRD of patterns for laser treated Ch/HAp/ZrO_2_ blends were analyzed in the 2θ angle.

**Figure 11:**
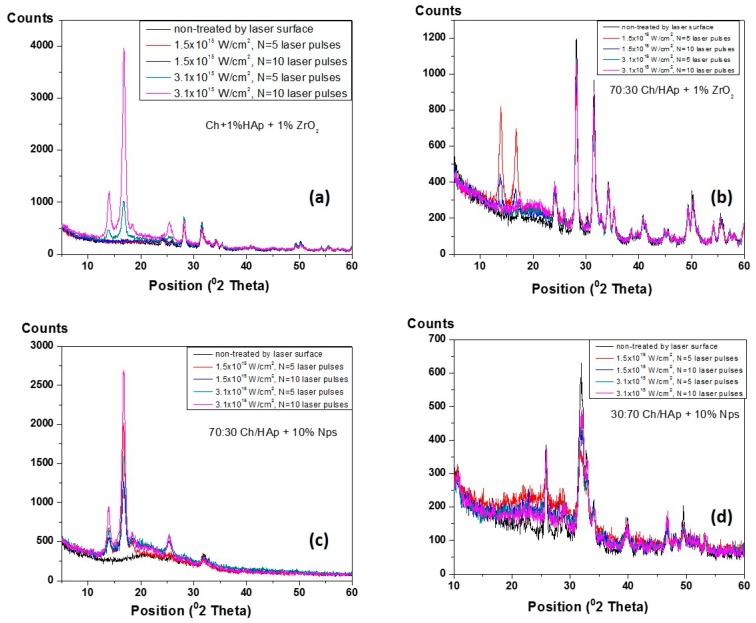
The XRD pattern of diverse composition of chitosan/ceramic blends before and after fs laser treatment. (**a**) Ch + 1% HAp + 1% ZrO_2_; (**b**) 70:30 Ch/HAp + 1% ZrO_2_; (**c**) 70:30 Ch/HAp + 10% Nps; (**d**) 30:70 Ch/HAp + 10% Nps.

In order to obtain basic information on the phase variations of the mineral components (hydroxyapatite and zirconia) before and after laser processing of synthesized composites, a series of XRD measurements were performed (Figure 11). The detected base lines of diffractogram occurred at angles 2θ = 14°, 17°, 19°, 25°, 28°, 31°, 34°, 40°, and 50° for each represented sample. The observed XRD peaks around 14–20° were attributed to the intercalated layers of the chitosan components [44]. The increased sharpness, intensities, and decreased broadness of mentioned peaks could be explained by the evolution in crystal sizes of the hydroxyapatite component [44,45]. Generally, the main differences in the XRD patterns depend on the constituent elements of the scaffold, and they are related to the maximum intensity variations in correspondance to the laser intensity and increase in the number of applied pulses, respectively. The main constituents for all scaffold variations must be taken into consideration. It was observed that the peak assigned to chitosan disappeared, and peaks attributed to the hydroxyapatite appeared more distinguished. These results indicated that HAp addition decreased the crystallinity of chitosan (because it dissolved in acetic acid), while chitosan had no influence on the hydroxyapatite crystallinity structure [46]. On XRD diagrams of composite films, the presence of chitosan peak was notable. However, due to the overlap with the spectrum of HAp, its identification was dificult but was determined by the other performed analysis (FTIR). Less pronounced HАp peaks occurred in the matrices with compositions of 70:30 Ch/HAp + 10% AgNps (Figure 11c),which could be explained by the minor amount of the HAp element present in the matrix, and was the expected result.

### 3.7. Cellular Affinity of Chitosan Ch/Hydroxyapatite (HAp)/ZrO_2_ Composite Films

**Figure 12:**
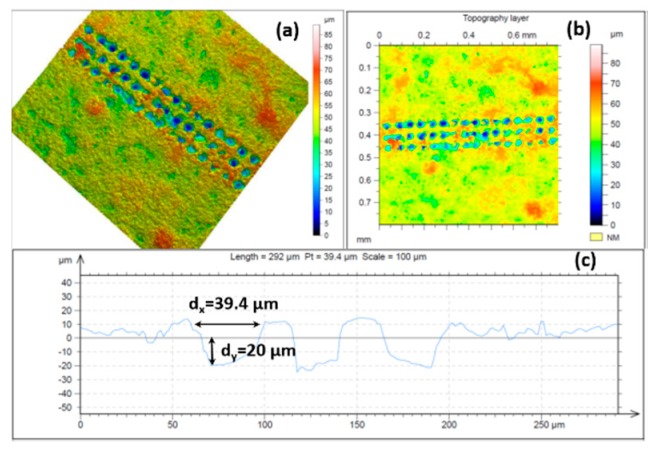
Confocal images of chitosan/ZrO_2_/HAp thin film irradiated at λ = 800 nm, at *I* = 3.1 × 10^15^ W/cm^2^, τ = 130 fs, *N* = 1; (**a**) 3-D topography image, (**b**) topography image; (**c**) cross section of the 3-D reconstructed image.

Cellular interaction with surfaces is a main issue that must be considered in the choice of biomaterials. The regulation of cell adhesion on the extracellular matrix (ECM) is governed by molecular mechanisms that provide a connection between the matrix and cell cytoskeleton. ECM topography is an important factor that influences the orientation of cell migration. When cells interact with designed matrices from diverse biomaterials, they gain a certain extracellular physicochemical signal from the topography and chemistry of the implant surface and consequently react. In this study, surface properties of chitosan/ZrO_2_/HAp composite films, specifically surface morphology and chemistry, were examined. The fabricated grooves on the composite thin layers onto which both types of cells were seeded were created in specific conditions that did not initiate material ablation, namely, *N* = 1, *I* = 3.1 × 10^15^ W/cm^2^ (Figure 6a,b). The grooves we obtained as a result of laser assisted modification were with fixed parameters. The estimated width and depth of the produced patterns, measured with confocal microscopy, were d_x_ ≈ 40 µm and d_y_ ≈ 20 µm, respectively (Figure 12).

Their influence on MC3T3 osteoblasts and adipose derived stem cells (ADSC stem cells) adhesion, proliferation, and orientation were also estimated. It was found that both types of cells strongly responded to microscaled surface topographic structures (Figure 6) of thin composite chitosan/HAp/ZrO_2_ composite films with diverse percentages of the main constituents.

**Figure 13:**
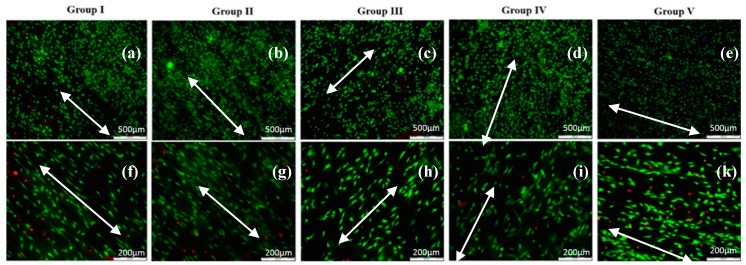
Fluorescence images of live-dead staining of mouse calvaria osteoblasts (MC3T3) cell alignment after 1-day culture on diverse blends of fs laser modified Ch/HAp/ZrO_2_ topographies (**a**–**e**). Close up images of the small protrusions of MC3T3 cells aligned to the laser created grooves on Ch/HAp/ZrO_2_ (**f**–**k**); white arrows show the direction of surface patterning and thus the direction of the grooves, which coincided with that of the cells.

On all types of laser modified thin composite films (Ch/1% HAp/1% ZrO_2_; 70% Ch/30% Hap + 1% ZrO_2_; 70% Ch/30% Hap + 10% Np; 30% Ch/70% Hap + 10% Np, 70%/30% Ch/HAp), the MC3T3 osteoblasts and ADSC stem cells attached to material surfaces exhibited significant orientation toward laser created stripes when compared to the non-modified parts of the surface. The micrographs of MC3T3 cells shown in Figure 13 were cultured on composite thin films and monitored after the first day.

**Figure 14:**
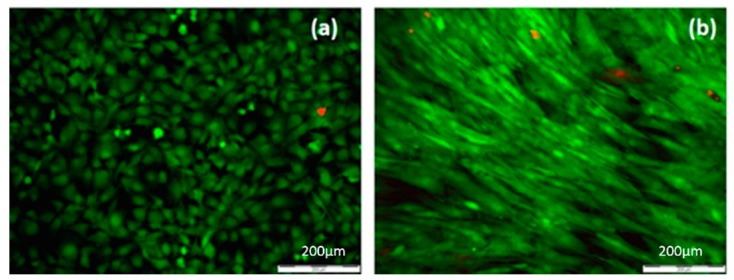
Fluorescence microscopy image of: (**a**) MC3T3 osteoblast cultured on non-treated surface for chitosan/ceramic composite film, (**b**) adipose derived stem cells (ADSC) cultured on control surface without laser treatment.

On the surface of the composite Ch/HAp/ZrO_2_ thin films control (Figure 14a), the MC3T3 osteoblast cells exhibited a bipolar shape and began to congregate in the form of clusters. On the laser treated part of the samples surface, the MC3T3 cells exhibited an extended shape directed towards the created microgrooves (Figure 13).

**Figure 15:**
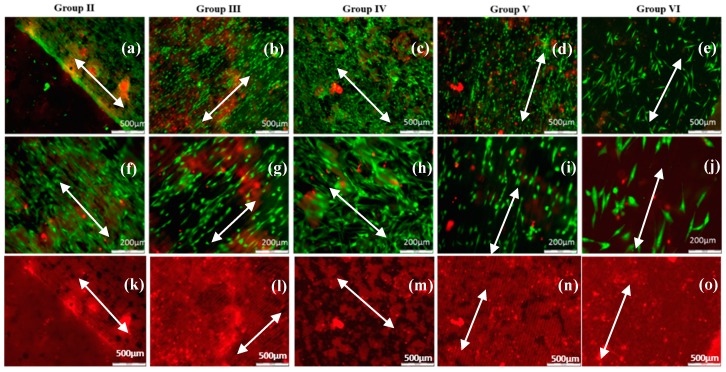
Fluorescence microscopy image of live-dead staining of ADSC stem cells distribution cultured for 6 days on Ch/HAp/ZrO_2_, fs laser modified in the form of stripes, thin composite films scale bar 200 µm (**a**–**e**). Close up images of ADSC stem cells orientation along structured surfaces, scale bar 500 µm (**f**–**j**), red fluorescent background images clearly show the lines/grooves on the samples (**k**–**o**); white arrows show the direction of surface patterning and the direction of the grooves, which coincided with that of the cells.

For all five groups (30% Ch/70% HAp, 70% Ch/30% HAp, 70% Ch/30% Hap + 1% ZrO_2_, 70% Ch/30% Hap + 10% Np, 30% Ch/70% Hap + 10% Np) of modified composite thin films, ADSC stem cells exhibited an elongated shape and grew as an adherent spreading monolayer along the laser formed grooves (Figure 15). Monitoring of ADSC stem cells on all substrates was performed after six days culture time. The enhanced orientation could be observed on most of the composite thin modified films in relation to the control (Figure 14b).

The cellular attachment to the ECM is a major factor in controlling important cellular processes such as adhesion, migration, proliferation, and differentiation. The laser created micropatterns of all samples induces the reorganization of cells movement that affects the cells cytoskeleton, consequently elongating its shape. Terakawa [47] made an extended review in which cell adhesion and behavior on the laser-irradiated surface of biodegradable polymers is described in detail.

All cells on the samples showed a high viability (Figure 13 and Figure 15) and were comparable to the cells seeded on the control tissue culture polystyrene (Figure 14). The results from the live/dead cell staining (Figure 13 and Figure 15) showed that the viable cells (green) were much more than the dead (red stained) ones and was indicative of the influence of the laser modification on the cellular properties. Both types of cells seeded onto the groove-like patterns demonstrated very high cell viability. Almost no dead cells were present. After a further culture period of six days (Figure 15a–j), the ADSC stem cells proliferated well on the groove-like patterns. This confirmed that the laser treated part of the samples surface created even better conditions for cells proliferation on the “bone mimic medium”. This further supports and develops previous research of the group [33,34]. The results obtained are of great importance in tissue engineering due to the requirement of a suitable microstructured matrix, which triggers the development of a large number of oriented cells for bone tissue repairing.

## 4. Discussion

It was found that implant surface properties affect primary stability and influence mechanical bonding with the tissue environment. Different studies have reported on the major role of tissue-to-implant adhesion for achieving primary stability. This property can be improved through implant surface processing [47].

Previous studies have been performed on surface processing by utilizing other laser sources and chemical methods, which have drawbacks related to collateral damage and side effects affecting the biocompatibility of the biomaterial [6,17]. The applicability of lasers working in different nanosecond or microsecond pulse durations for material processing is associated with the development of heat affected zones, melting, and evaporation of the material [48,49].

In contrast, the femtosecond laser modification method has significant advantages related to material treatment and ablation with high precision without the development of thermal damage or microcracks formation. Malinauskas et al. successfully applied direct laser writing (DLW) ablation to modify the surfaces of the PLA structures by fabrication of 3D microporous structures with functionalization properties. In their studies, a three-dimensional printing (3DP) approach was applied for the fabrication of macrostructures with internal 3D microstructure. The DLW via ablation was employed to modify the surfaces, hole drilling, and outer geometry shaping of the whole objects and create surface textures on micro- and nano-scales [50].

The high peak power of femtosecond pulses allows it to locally melt, vaporize, or ablate the material. The process develops with “cold ablation” [51,52,53,54].

Interaction in the femtosecond time domain is based on the nonlinear processes of light absorption and ionization [51,54].

In this research, a series of composite thin films was prepared by blending chitosan with hydroxyapatite, Zirconia (ZrO_2_), and Ag nanoparticles in various proportions, and the surface morphology and chemistry of these composite films were systematically examined.

After the incorporation of a ceramic component with the main constituent, the surfaces of composite films showed a granule-like topography. These observations in surface topography could be assigned to the different crystallization process between chitosan and ceramic particles. The chitosan matrix possesses intermolecular and intramolecular hydrogen bonds. These bonds are involved in the crystallization process by forming particle crystals [55].

The laser treatment at 800 nm laser radiation produced a clean surface modification at a lower intensity range without the formation of melting and cracks. It was noted that the surface treatment of chitosan composite samples by fs laser irradiation produced adequate structuring from the point of view of surface topography. For most types of materials, melting occurred at very high intensities near the peak of the Gaussian beam. Laser modification of biopolymer/ceramic blends initiated a process of morphological surface changes in very low levels of laser intensities. The damage induced by femtosecond laser pulses at intensities exceeding 3.1 × 10^15^ W/cm^2^ resulted in composite material ablation. Increment of intensity and number of pulses triggered the development of microscale rough structures. As intensity increased further, the formation of deeper craters on the biopolymer and the destruction of polymer/ceramic matrices were observed. In the case of biopolymer/ceramic blends, signs of open porosity were detectable, which suggested the initiation of a bubble formation mechanism attributed to liquid–vapor instability.

From the AFM results of pure chitosan film treatment with diverse expositions to selected number of applied laser pulses (*N*), it was estimated that by increasing *N* and laser intensity, the roughness parameter (*R*_rms_) increased proportionally. The overall roughness was a consequence of the superposition of the created patterns (depending on the set condition for overlapping between separate laser shots) and additional side effects like cracks or pores formation. It was found that the laser parameters interplay (intensity and number of pulses) affected surface roughness [54]. The tunability of laser processing via variation of laser intensity and number of pulses had a joint effect on the morphology of the modified material. In order to obtain porous patterns, both the intensity and the number of pulses had to be kept (in the case of pure chitosan and also for its ceramic blends) in its low range. SEM, FTIR, and XPS analysis proved that this regime led to defined structuring without drastic alteration of chemical composition. Ideal porous modifications could be achieved by irradiation with a low number of pulses (*N* = 1 and 2) for deeper patterns and an increase of *I* and *N* led to the disruption in the quality of the structures.

Different reports have indicated that the surface properties drastically influence cell behaviors [33,34,35,47,56,57,58,59].

The examination of the influence of laser created surface patterns on composite films on two types of cells (MC3T3 osteoblasts and ADSC stem cells) provides a preliminary model for cell-interface studies. The monitoring of cellular adhesion and proliferation on all six groups of composite films was achieved. The results from cellular studies on fs laser modified films showed indicative proliferation and orientation of MC3T3 osteoblasts and ADSC stem cells compared to the non-treated control. One possible explanation for these discoveries was that the substrate surfaces had increased roughness that mimicked, in part, the microstructure of the native extra-cellular matrix (ECM). In the human body, the ECM serves as a support to cells and guides their mobility [60].

Moreover, the influence of surface patterning on protein adsorption [47] must also be explored. It has been reported that protein absorption plays a main role in cellular adhesion, which is explained in detail in the review of Khalili and Ahmad [60].

Another property that has been found to drastically affect cell adhesion is the presence of microporosity in biocomposite scaffolds [61], which is an important factor in allowing cellular infiltration and proliferation inside a volume. The natural hierarchical architecture of bone ranges from pores of around 1 μm (interaction with proteins) to 1 to 20 μm (cellular in-growth), 100 μm and up to more than 500 μm (for cellular growth and bone in-growth). With laser-assisted technology, control of porosity via the selection of a number of applied laser pulses and intensity can be achieved and adapted for implant materials.

## 5. Conclusions

In summary, femtosecond laser material processing represents a fast and precise method for initiating controlled morphological changes at the micrometric scale on the surface of chitosan and its ceramic blends. Enhancement of physicochemical and morphological properties of the chitosan and its ceramic composite blends were studied. The interplay between the applied laser parameters and the treatment conditions gives diverse structural alterations. These studies show that the laser surface processing technique is an alternative method to conventional surface treatment techniques like sandblasting and acid etching because it permits easy control of the roughness without additional chemical contamination, which in turn helps to enhance cellular adhesion. Tuning laser parameters permits close control over pattern type and properties like roughness and depth.

## Figures and Tables

**Table 1:** Classification of the prepared samples compositions: Ch-chitosan, HAp-hydroxyappatite, Ag Nps-silver nanoparticles.

Group Number	Material
**I**	Ch/1% Hap/1% ZrO_2_
**II**	70% Ch/30% Hap + 1% ZrO_2_
**III**	70% Ch/30% Hap + 10% AgNps
**IV**	30% Ch/70% Hap + 10% AgNps
**V**	70% Ch/30% HAp
**VI**	30% Ch/70% HAp

**Table 2:** The chemical bonds detected by FTIR spectrogram of composite chitosan/ceramic thin films.

Wave Number (cm^−1^)								
Samples	P=O	PO_4_^3−^	CO_3_^2−^	–CH_3_; –CH_2_	C=O; =N–H I/II	=N–H III	C–H “backbone”	–OH
Ch + 1% Hap + 1% ZrO_2_	1130–1150	600–500; 1032	2363	1400	1650	1267	2900–2880	3400–3000
70% Ch/30% Hap + 1% ZrO_2_	1130–1150	600–500; 1032	2363	1400	1650	1267	2900–2880	3400–3000
